# Subcutaneous Inoculation of *Echinococcus multilocularis* Induces Delayed Regeneration after Partial Hepatectomy

**DOI:** 10.1038/s41598-018-37293-0

**Published:** 2019-01-24

**Authors:** Shadike Apaer, Tuerhongjiang Tuxun, Heng Zhang, Amina Aierken, Tao Li, Jin-Ming Zhao, Hao Wen

**Affiliations:** 1 State Key Laboratory of Pathogenesis, Prevention, Treatment of High Incidence Diseases in Central Asia, First Affiliated Hospital of Xinjiang Medical University, Urumqi, China; 2 Department of Liver Transplantation & Laparoscopic Surgery, Digestive & Vascular Surgery Center, First Affiliated Hospital of Xinjiang Medical University, Urumqi, China; 3 WHO Collaborating Center for Prevention and Care Management of Echinococcosis, First Affiliated Hospital of Xinjiang Medical University and Xinjiang Centers for Disease Control, Urumqi, China; 4 Department of Surgery, ZhuJiang Hospital of Southern Medical University, Guangzhou, China; 5 Health Management Center, Xinjiang Medical University, Urumqi, China

## Abstract

Alveolar echinococcosis (AE) is caused by the larval stage of *echinococcus multilocularis* (*E*. *multilocularis*), and hepatectomy is the main modality in hepatic AE patients. Liver regeneration after partial hepatectomy (*PHx*) in such patients is challenging, and further investigation is needed. Thus far, knowledge regarding the possible impact of *E*. *multilocularis* on liver regeneration after *PHx* is limited. Herein, a subcutaneous infection model of *E*. *multilocularis* was developed in C57 BL/6 mice, and after 3 months, *PHx* was performed. Plasma and liver samples were harvested under inhalational isofluorane (2%) anaesthesia at designated post-*PHx* time points (0, 24, 48, 96 and 168 h). The parameters included the future remnant liver/body weight ratio (FLR/BW), liver function tests (AST and ALT) and related cytokines (TNF-α, IL-6, Factor V, HMGB1, TGF-β, TSP-1, and TLR4) and proteins (MyD88 and STAT3). To assess the proliferation intensity of hepatocytes, BrdU, Ki67 and PAS staining were carried out in regenerated liver tissue. The FLR/BW in the infected group from 48 h after surgery was lower than that in the control group. The BrdU positive hepatocyte proportions reached their peak at 48 h in the control group and 96 h in the infected group and then gradually decreased. During the first 48 h after surgery, both the AST and ALT levels in the infected group were lower; however, these levels were altered from 96 h after surgery. In the infected group, the concentrations and mRNA expression levels of the pre-inflammatory cytokines TNF-α and IL-6 demonstrated a delayed peak. Moreover, post-operatively, the TGF-β and TSP-1 levels showed high levels in the infected group at each different time-point compared to those in the control group; however, high levels of TGF-β were observed at 96 h in the control group. The MyD88 and STAT3 protein expression levels in the infected group were markedly higher than those in the control group 96 h after surgery. Delayed liver regeneration after *PHx* was observed in the C57 BL/6 mice with the subcutaneous infection of *E*. *multilocularis* in the current study. This phenomenon could be partially explained by the alteration in the pro-inflammatory cytokines in the immunotolerant milieu induced by chronic *E*. *multilocularis* infection.

## Introduction

Alveolar echinococcosis (AE), which is caused by the larval stage of the metacestode *Echinococcus multilocularis* (*E*. *multilocularis*), continues to be a major public health issue worldwide, especially in China, Central Asia, the Middle East and some parts of Europe^1,2^. AE lesions primarily target the liver and present as “cancer like” infiltrative growths. Surgical resection in conjunction with post-operative albendazole therapy is conclusive; however, the available data show that only a few patients benefit from radical resection^3^. The intensive vascular involvement and insufficient remnant liver (RL) volume are the most common factors influencing the treatment paradigm^3–5^.

For decades, advancements in surgical techniques and adjuncts have resulted in improved clinical outcomes in hepatic AE patients. Nevertheless, post-operative hepatic dysfunction is still a real challenge^6,7^. Hepatocyte hypertrophy in the unaffected lobe in hepatic AE patients was first reported four decades ago^8,9^. A clinical cohort of 81 end-stage hepatic AE patients at our centre showed a significant increase in the AE lesion-free liver lobe. This phenomenon was explained by the portal flow reallocation after the obliteration of the portal vein in the diseased lobe and possible circulating pro-inflammatory cytokines induced by the AE infection^5,10,11^. However, only a few studies reported preliminary results regarding parasitic infections and liver regeneration in murine models after partial hepatectomy^12,13^. In addition, neither clinical nor experimental studies focusing on the mechanism have been reported.

In the current study, subcutaneous inoculation of *E*. *multilocularis* 3-month prior to *PHx* was performed. The potential influence of *E*. *multilocularis* infection on liver regeneration in a murine model was assessed and discussed.

## Results

### Evaluation of subcutaneous *E*. *multilocularis* infection model

**Figure 1:**
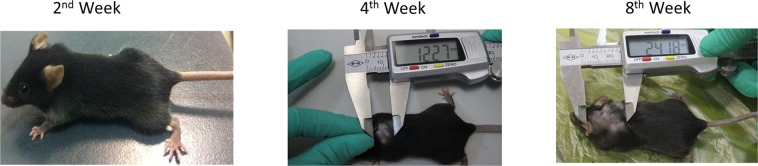
Evaluation of the subcutaneous *E*. *multilocularis* infection model. Diameter of *E*. *multilocularis* lesions on the cervical back was measured with Vernier callipers at different time points after infection.

The total success rate of the subcutaneous *E*. *multilocularis* infection model was 82% (41/50). The size of the *E*. *multilocularis* lesions was measured at the cervical back with Vernier callipers at various time points after infection (Fig. 1). The maximum/minimum diameter was 24.80 mm × 22.60 mm and 7.30 mm × 7.10 mm, respectively, with a mean of 14.46 mm × 10.98 mm.

### Survival and liver regeneration assessment

**Figure 2:**
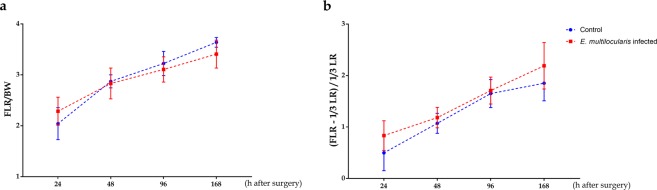
Kinetic features of remnant liver (LR) mass regeneration after *PHx*. (**a**) Future liver remnant mass to body weight ratio (FLR/BW) indicating restoration of liver mass was measured at designated time points. Between 48 and 168 h after surgery, *E*. *multilocularis* infection leads to decreased liver restoration. (**b**) [(FLR-1/3 LR)] to 1/3 LR ratio was calculated, and the data presented a slightly high restoration ability of 1/3 LR in the *E*. *multilocularis* infection mice at all indicated time points compared with that in the control mice.

The overall survival rate after surgery was 100%. The liver mass recovery, future liver remnant mass and body weight ratio (FLR/BW), (FLR - 1/3 LR) and 1/3 LR ratio after surgery were calculated at designated time points in both groups (n = 8/group per time point). After 24 h, FLR/BW in the infected group was higher than that in the controls; in contrast, after 48 h, the FLR/BW ratio in the controls exceeded that in the infected groups (Fig. 2a). The (FLR - 1/3 LR) and 1/3 LR ratio in the infected group were higher than those in the control group at all designated time points, albeit without a statistically significant difference (*p* > 0.05, Fig. 2b).

### Liver function test

**Figure 3:**
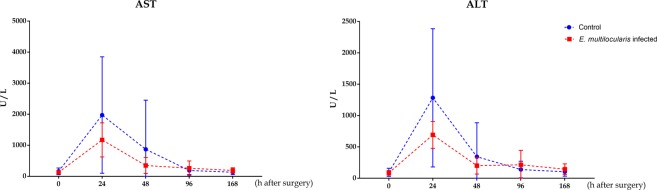
Plasma transaminases in the two groups at the indicated time points. Plasma aspartate aminotransferase (AST) and alanine aminotransferase (ALT) concentration levels during the first 48 h after surgery were reduced and subsequently gradually elevated in the *E*. *multilocularis* infected mice compared to those in the control mice. The data represent the *Mean (X̅)* ± *Standard deviation* (*SD*).

The perioperative plasma levels of AST and ALT did not differ between the two groups at each time point. Dynamically, the AST and ALT levels peaked 24 h after surgery in both groups and gradually decreased at 168 h in both groups. Notably, their levels in the control group were higher at all-time points, except for 96 and 168 h after surgery, than those in the infected group; however, no statistically significant differences were observed (*p* > 0.05, Fig. 3).

### Factor V, HMGB1, IL-6, TGF-β and TSP-1 concentrations in plasma

The Factor V concentrations in the infected group were markedly higher than those in the controls prior to surgery (*p* < 0.01). However, the Factor V concentrations were markedly increased in the control group 24 h after surgery (*p* < 0.01). In contrast, at 96 and 168 h, the concentrations in the infected group were statistically significantly higher than those in the controls (*p* < 0.01, *p* < 0.001).

The plasma concentrations of HMGB1 in the infected group were markedly higher than those in the controls prior to surgery, but no statistically significant differences were observed (*p* > 0.05). However, the HMGB1 concentrations were extremely and slightly increased in the controls at 24 h and 48 h, respectively (*p* < 0.05, *p* > 0.05). In contrast, at 96 h and 168 h, the HMGB1 concentrations in the infected group were statistically significantly higher than those in the controls (*p* < 0.01, *p* < 0.05).

The concentrations of IL-6 in the controls were increased after surgery and higher than those in the infected group at 24, 48 and 96 h, but statistical significance was found only at the 24 h after surgery time point (*p* < 0.01). In contrast, the concentrations were higher in the infected group prior to surgery and 168 h after surgery, and statistical significance was only observed prior to surgery (*p* < 0.05).

The plasma TGF-β concentrations in the infected group prior to surgery and 96 h after surgery were slightly higher than those in the controls (*p* > 0.05). However, at all remaining time points, the concentrations in the controls were slightly higher than those in the infected group (*p* > 0.05).

**Figure 4:**
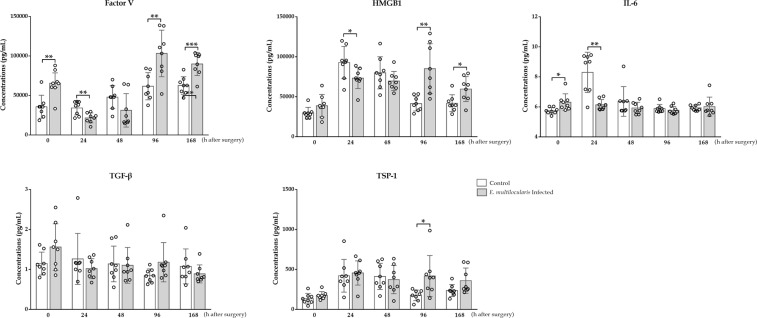
Cytokine concentrations in peripheral plasma at designated time points. ELISA results demonstrating that *E*. *multilocularis* infection could suppress the VEGF, HMGB1 and IL-6 concentrations at 24 h, and these levels were increased 96 and 168 h after surgery. TGF-β and TSP-1 levels 96 h after surgery in the *E*. *multilocularis* infected group were significantly higher than those in the control group. The data are expressed as the *Median (Interquartile)*, and statistical significance is marked as follows: ^*^p ≤ 0.05, ^**^p ≤ 0.01 and ^***^p ≤ 0.001.

The TSP-1 concentrations in the infected group were higher than those in the controls at all-time points, except for 48 h, and statistical significance was found only at 96 h after surgery (*p* < 0.05). At 48 h after surgery, the concentrations in the controls were higher than those in the infected group, albeit no statistical significance was observed (*p* > 0.05, Fig. 4).

### Histological examination and BrdU labelling

**Figure 5:**
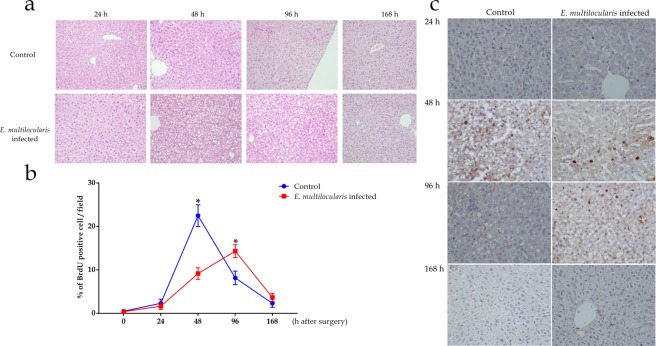
Morphological changes and DNA replication of hepatocytes were detected using haematoxylin and eosin (H&E) and immunohistochemistry techniques. (**a**) Severe hepatocyte oedema, balloon-like degeneration, and even steatosis were observed, especially in the *E*. *multilocularis* infected mice (original magnification 200×). (**b**,**c**) Significantly higher BrdU-positive hepatocyte proportions were observed in the controls compared to those in the infected group 48 h after surgery; in contrast, at 96 h, the proportions in the infected group were significantly higher than those in the control group (original magnification: 400×; *p* < 0.05). The data represent the *Mean* (*X̅*) ± *Standard deviation*.

The morphological changes in the hepatocytes in both groups included oedema and balloon-like degeneration at 24 h, and severe steatosis developed 48 h after surgery. The severity of steatosis in the *E*. *multilocularis* infected group at 48 h and 96 h after surgery was especially higher than that in the controls; then, the normal morphology of the hepatocytes was gradually restored (Fig. 5a).

BrdU positive areas were mainly observed in the hepatocyte nuclei (Fig. 5b), and a slight increase in the BrdU positive cell proportion was observed in both groups 24 h after surgery. The BrdU positive cells in both the control and infected groups peaked with high levels 48 h and 96 h after surgery, respectively. The mean BrdU positive cell counts were tremendously higher in the control group than those in the infected group 48 h after surgery; in contrast, the counts were significantly higher in the infected group 96 h after surgery (*p* < 0.05). The numbers of BrdU positive hepatocytes were similar between the two groups 168 h after surgery (Fig. 5c).

**Figure 6:**
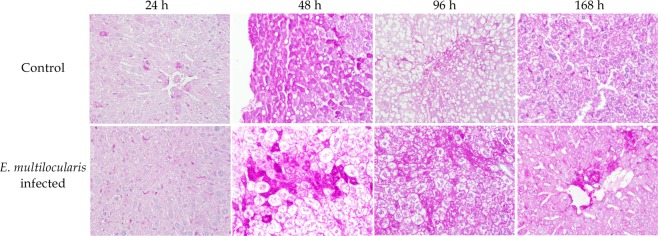
Periodic acid–Schiff (PAS) staining in liver tissues. Representative liver tissue sections showing weak positive cells 24 h after surgery in both the control and *E*. *multilocularis* infected groups. Considerable glycogen staining was observed in the control group at 48 h; apparently increasing levels and peak levels were observed at 48 h and 96 h after surgery in the infected group, respectively (original magnification: 200×).

Relatively weak Periodic acid–Schiff (PAS) staining was detected in both groups 24 h after surgery; however, considerable numbers of glycogen positive cells were observed 48 h after surgery in the controls, and then, the number of these cells gradually decreased. Apparent increasing levels of glycogens were observed 48 h after surgery; these levels peaked at 96 h and remained high until the end point (Fig. 6).

The CD31 staining results revealed a significantly higher microvessel density in the control mice 48 h after surgery, which subsequently gradually decreased. In the infected mice, a sharp increase in blood vessels was observed at 48 h, and a summit point was reached 96 h after surgery (Supplementary Fig. S1). Both groups displayed extremely weak Ki67 levels that could not be observed (data not shown).

### TNF-α and IL-6 mRNA expression levels in regenerated liver tissues after *PHx*

**Figure 7:**
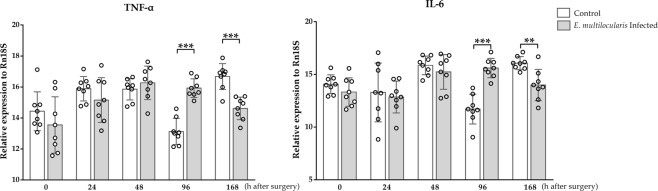
Dynamic changes in TNF-α and IL-6 mRNA expression levels. qRT-PCR results demonstrated that *E*. *multilocularis* infection suppresses TNF-α and IL-6 mRNA expression levels prior to surgery and 24 h and 168 h after surgery; however, these levels were significantly increased at 96 h. The data are expressed as the *Mean* (*X̅*) ± *Standard deviation* (*SD*).

The relative mRNA expression levels of TNF-α in the hepatic tissues in the control group were slightly higher than those in the infected group prior to surgery. After 24 and 168 h, the expression levels in the control group were higher than those in the infected group, and statistical significance was found 168 h after surgery (*p* < 0.001). At 48 and 96 h after surgery, the TNF-α expression levels in the infected group were higher than those in the control group, and statistical significance was found at 96 h (*p* < 0.001). The IL-6 mRNA levels in the hepatic tissues in the control group were slightly higher than those in the infected group prior to surgery. At 24, 48 and 168 h after surgery, the IL-6 expression levels in the control group were higher than those in the infected group, and statistical significance was found 168 h after surgery (*p* < 0.01). The IL-6 mRNA expression level in the infected group was significantly higher than that in the control group 96 h after surgery (*p* < 0.001, Fig. 7).

### TLR4, MyD88, TSP-1 and TGF-β mRNA expression levels in regenerated liver tissues after *PHx*

**Figure 8:**
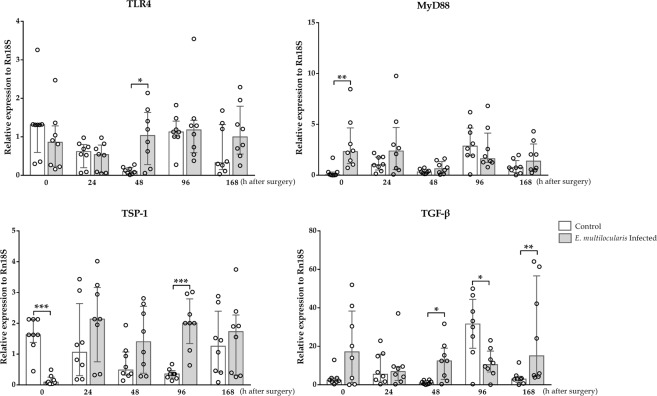
TLR4, MyD88, TSP-1 and TGF-β mRNA expression levels in liver tissues after *PHx*. TLR4 and MyD88 mRNA expression levels were increased 48 h after surgery. TSP-1 and TGF-β levels after surgery were increased compared with those in the controls. The data represent the *Mean* (*X̅*) ± *Standard deviation* (*SD*).

The TLR4 mRNA expression levels in the regenerated liver in the infected group were significantly higher than those in the control group 48 h after surgery (*p* < 0.05). The MyD88 mRNA expression levels in the infected group were significantly higher than those in the control group prior to surgery (*p* < 0.01). The TSP-1 mRNA expression levels in the control group were significantly higher than those in the infected group prior to surgery (*p* < 0.001); in contrast, the expression levels in the regenerated liver tissues 96 h after surgery were significantly higher in the infected group compared with those in the control group (*p* < 0.001). The TGF-β mRNA expression levels in the infected group were significantly higher than those in the control group 48 h and 168 h after surgery (*p* < 0.05, *p* < 0.01); however, these levels were significantly lower in the infected group 96 h after surgery (*p* < 0.05). The detailed dynamic alterations in the abovementioned mRNA expression levels are shown in Fig. 8.

### MyD88 and STAT3 protein expression levels in regenerated liver tissues after *PHx*

**Figure 9:**
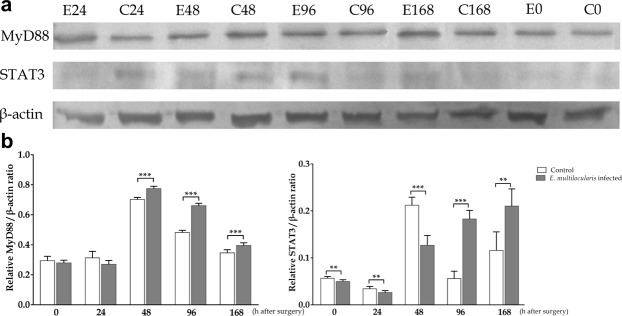
Western blot analysis of MyD88 and STAT3 protein expression levels. Representative images (**a**) and densitometric quantification (**b**) of the expression of MyD88 and STAT3 as assessed by a Western blot analysis of the total protein liver extracts at indicated times after surgery. *E*. *multilocularis* infection suppresses MyD88 expression levels prior to surgery and 24 h after surgery; however, these levels were significantly increased at all other time points (*p* < 0.001). At 24 h and 48 h after surgery, low levels of STAT3 were observed, and higher expression levels were observed at 96 and 168 h with statistical significance. β-actin was used as a loading control, and the data are expressed as the *Mean* (*X̅*) ± *Standard deviation* (*SD*).

The MyD88 protein expression levels in the control group prior to surgery and 24 h after surgery were slightly higher than those in the infected group (*p* > 0.05). At 48, 96 and 168 h after surgery, the MyD88 protein levels in the infected group were significantly higher than those in the controls (*p* < 0.001). The STAT3 protein expression levels in the control group prior to surgery and at 24 and 48 h after surgery were markedly higher than those in the infected group (*p* < 0.01, *p* < 0.01, *p* < 0.001); in contrast, the levels in the infected group were significantly higher than those in the control group 96 and 168 h after surgery (*p* < 0.001, *p* < 0.01, Fig. 9).

## Discussion

In this study, *PHx* was performed in a mouse model of subcutaneous *E*. *multilocularis* infection to explore the alterations in circulatory and local related cytokines and their possible influence on the post-hepatic resection regenerative process. This study is the first to report that *E*. *multilocularis* infection could delay the remnant liver regeneration process and that the immune-tolerance milieu induced by *E*. *multilocularis* infection might be a determining factor during this process.

Based on both experimental and clinical studies, it is well-known that *E*. *multilocularis* infection could actively modulate the host immune system^14–16^. Among the modulators, pro- and anti- inflammatory cytokines play a vital role in parasite clearance and growth^17,18^. Type 1 helper T (Th1) cell immunity, which functions via its related effector cytokines TNF-α and IFN-γ, plays a protective role during the early stage, while regulatory cytokines, such as IL-10 and TGF-β, which are mainly secreted by Th2 subsets, are associated with infiltration of metacestodes and disease susceptibility. Our previous study showed that IL-6 and TNF-α are actively involved in disease progression and granuloma formation^19,20^. Increasing evidence suggests that Th17 and Treg cells are closely related to immune tolerance phenomenon during *E*. *multilocularis* infection^21^. More recently, we reported that IL-17 and IL-23 are associated with disease progression in both experimental and clinical studies^14,22^.

The liver has a high regenerative capacity, and the immune patterns are thought to perform pivotal functions during the initiation and modulation process after injury or resection^23^. Evidence from studies involving humans or mammals suggests that immunological factors, including a series of extrinsic and intrinsic cytokines (i.e., TNF-α, IL-6, etc.), multiple cell-signalling pathways (i.e., LPS/TLRs/MyD88) and their downstream cascades (i.e., STAT3) interact and play a critical role in the liver regeneration process^24,25^. Both peripheral and regional remnant hepatic TNF-α levels are elevated, resulting in the further activation of NF-κB and IL-6 induction after *PHx*^24,26,27^. The IL-6/IL-6R complex could activate STAT3, further stimulate liver cells, and enhance the hepatocyte protection ability and survival^28–30^. Both IL-6 and TNF-α knockout mice exhibit severe deficits in liver regeneration after *PHx*^29–32^. In the present study, the rapid decrease in the TNF-α and IL-6 relative expression levels at 96 h may be due to the beginning of the termination phase of the liver regeneration process.

Since an interaction exists between cytokine alterations after *E*. *multilocularis* infection and liver regeneration, we developed a model to investigate whether *E*. *multilocularis* infection has any impact on liver regeneration. Interestingly, the results demonstrated delayed regeneration after *PHx*. To the best of our knowledge, the first report investigating parasitic infection and liver regeneration was based on Teixeira *et al*.*’*s^33^ pioneering research. Costa *et al*.^34^ postulated that the pro-inflammatory cytokines TNF-α and IFN-γ, which are produced by Th1 cells during the acute phase of *Schistosoma mansoni* infection, may contribute to the enhanced liver regeneration after *PHx*. In our clinical practice, based on more than 400 AE hepatic resections, hypertrophy of the unaffected liver parenchyma occurs frequently. This finding might be partially attributed to the rearrangement of portal blood flow caused by parasitic portal obliteration in the diseased lobe. Previous studies conducted by our team have shown extensive liver fibrosis in both human and murine echinococcosis, but the exact mechanism has not been clearly uncovered to date^35–37^. Lin *et al*.^35^ observed heavy liver fibrosis in tissues near AE lesions compared with tissues in distant areas, suggesting that *E*. *multilocularis* infection might induce or accelerate liver fibrosis. These authors concluded that TGF-β plays a significant role leading to immune tolerance in this immune-pathologic injury. Simultaneously, Zhang *et al*.^38^ observed distinct anti-apoptotic and hepatocyte proliferative characteristics during the early and middle stages of *E*. *multilocularis* infection *in vivo*.

The TLR and MyD88-mediated pathways are responsible for the recognition of and responsiveness to conserved pathogen-associated molecular patterns (PAMPs), such as *E*. *multilocularis* infections, and most importantly, these pathways could also influence subsequent acquired immunity by preferentially inducing Th1 cell-derived responses^39^. Our previous studies observed both regional and systematic elevation in TLRs in patients with hepatic AE, suggesting that TLRs actively interact and may play a significant role during infection^14^. Intriguingly, the current study demonstrated a relatively delayed (48 h after surgery) increase in the TLR4 mRNA expression levels in the regenerating liver tissues. In addition, the high MyD88 mRNA expression levels were altered at 96 h but remained significantly higher at the other time points, and compared with the control group, the protein levels were elevated after 48 h in the *E*. *multilocularis* infected group (Figs. 8–9). Subsequently, the TLR and MyD88 pathways in the non-parenchymal liver cells mediated the induction of the Th1 cell type cytokine TNF-α, which is believed to play a pivotal promotive role during the priming and progressing stages of liver regeneration; however, in our results, their patterns were restricted during the priming phase (Fig. 7), which might be caused by the eradication of the Th2/Treg cell type predominant immune response during the *E*. *multilocularis* chronic phase.

TSP-1 is considered a negative regulator of liver regeneration^40^. Clinical observational studies have indicated the predictive potential of TSP-1 in liver dysfunction after hepatectomy^41,42^. Elevated circulatory TPS-1 levels are associated with delayed recovery and even poor outcomes in patients who underwent partial resection^43^. TSP-1 can convert latent TGF-β1 into biologically active TGF-β1, which is also called the TSP-1/TGF-β axis, contributing to the blockade of hepatocyte proliferation^40,42^. TGF-β mRNA has been shown to begin to release immediately after *PHx*, reach a plateau at approximately 24 h, and remain steady for at least 4 days after surgery^27,44,45^. Our data from the *E*. *multilocularis* infected mice revealed a blunt increase with a peak 96 h after surgery. Studies have shown that TGF-β can maintain hepatocytes in the G0 phase through an anti-growth factor effect. The elimination of TGF-β and its receptors accelerates DNA synthesis, further enhances regeneration and prolongs hepatocyte proliferation^46,47^. In the present study, consistent with above paradigm, the TGF-β mRNA levels in the liver tissues began to increase 24 h after surgery and continued to elevate until 48 h. Approximately 96 h after *PHx*, TGF-β was increased, especially in the non-infected mice and infected mice at 168 h, which may be indicative of the termination phase of liver regeneration with some delay in the latter group. However, the plasma TGF-β concentrations reached the peak levels in the non-infected mice; the highest delayed points were observed 96 h after surgery. We assume that the persistent stimulus and chronic inflammation responses caused by *E*. *multilocularis* infection may be responsible for this delayed “crest”.

Additionally, as an important chromatin protein, HMGB1 performs multiple functions. In the cell nucleus, HMGB1 facilitates the transcription of genes that could interact with transcription factors, such as NF-κB^48^. In injured tissues, HMGB1 can also trigger inflammation, which is often accompanied by tissue repair^49,50^. In the liver, HMGB1 is secreted by various types of immune cells and acts as a mediator cytokine of inflammation^51^. Studies have revealed that the above action of HMGB1 is often closely related to its binding or interactions with LPS-TLR4, further leading to TLR4 activation, and its binding MyD88 results in signal transduction and downstream cascades^52^. In the present study, the plasma HMGB1 levels were increased early after surgery in the control mice but exhibited a delayed peak at 96 h in the *E*. *multilocularis* infected mice.

Previous results from our basic and clinical studies have shown that during the late stage of *E*. *multilocularis* infection, the Treg/Th17 ratio was significantly elevated, resulting in a Treg-dominant suppressive immune response. After liver parenchymal resection, LPS could strongly stimulate Kupffer cells to commence the cascade responsible for regeneration. However, in *E*. *multilocularis* infection, LPS fails to “wake-up” the immune response because of the immune tolerance formed by the continuous metacestode stimulation. Consistent with our expectations, after *E*. *multilocularis* infection, the host Th2-type immune responses may attenuate Th1 cell related immune patterns and cytokine secretion and then weaken the regeneration ability. In addition, as shown in our and others’ previous reports, the late stage of AE is characterized by a Th17/Treg imbalance that plays crucial roles in the formation of the host’s immune tolerance. Thus, the decreased reactivity of the immune system to pathological stimulators (e.g., LPS) may be another potential reason for the delayed regeneration in the *E*. *multilocularis* infected subjects.

Although this study aimed to reveal the potential impact of *E*. *multilocularis* infection on post-operative liver regeneration, several inherent limitations should be considered. First, this study was based on a murine model; thus, the findings of the current study may not fully translate to human subjects. In addition, only MyD88 related liver regeneration was considered, and other important pathways might have been omitted in the current work and require further in-depth investigation.

## Conclusion

This study is the first to report the possible impacts of *E*. *multilocularis* infection on the liver regeneration process after *PHx*. The suppression of Th1 cell-related pro-inflammatory cytokines, such as TNF-α and IL-6, by the Th2-type cell immune patterns established during the chronic phase of *E*. *multilocularis* infection may be responsible for the delayed hepatocyte proliferation. Further investigations and vigorous ongoing efforts should be made to identify the more precise mechanisms of the liver regeneration process and its interactions with immunological conditions, which could be very helpful for providing effective clinical intervention methods to promote regeneration after hepatectomy and liver transplantation.

## Materials and Methods

### Ethical statements and animal treatments

All animal experiments and treatments were conducted under the strict guidelines and approval of the Institutional Animal Use and Care Committee of Xinjiang Medical University (Approval Number: 20150225-116). The 6- to 8-week-old female C57 BL/6 mice (weighing 18-21 g) were purchased from the experimental animal centre of Xinjiang Medical University. The mice were housed in a specific pathogen-free facility under 12:12 h light/dark cycles at room temperature and humidity with unlimited *ad libitum* access to water and standard animal food.

### Establishment of subcutaneous *E*. *multilocularis* infection model

Metacestode was isolated from intraperitoneally infected gerbils. Briefly, after grinding through 200 mesh steel strainers, the lesion tissues were washed several times with 0.9% normal saline (NS) under aseptic conditions. Then, 100 μL of NS suspension containing approximately 2000 vesicular cysts were prepared and subcutaneously injected into the mice. In the control animals, 100 μL of NS solution were used.

### Surgery

After an overnight fast, 2/3 *PHx* based on the classic model^53^ with slight modification was performed on days 90 (referred to as the late stage of *E*. *multilocularis* infection) under inhalational isofluorane (2%) anaesthesia (Supplementary Fig. S2). Then, 2.5–3.0 cm median laparotomy and separation of ligations were implemented. With ligation at the basis (close to the inferior vena cava) of the left lateral and median lobes, the above two lobes were removed, and the peritoneal cavity was closed with a single layer continuous suture. The mice were placed on an insulation blanket until full resuscitated from anaesthesia.

### Assessment of the rate of liver regeneration after *PHx*

The resected liver mass was weighed to assess FLR/BW. Then, 2 h before sacrifice, the mice were treated with *i*.*p*. bromodeoxyuridine (BrdU, 50 mg/kg in a 0.2% solution of phosphate-buffered saline, PBS). At the indicated time points after surgery (0, 24, 48, 96 and 168 h), plasma and liver tissue samples were harvested under the same inhalation anaesthesia method described above for the surgery process. To observe the regenerative intensity of LR, 1/3 LR was calculated based on the FLR mass, and the (FLR-1/3 LR) and 1/3 LR ratio were assessed.

After fixation in 4% neutral buffered formalin for at least twenty-four hours and paraffin-embedding, samples of representative liver tissues were sectioned into 3–4 μm slices using a microtome (Leica, Japan). The slices were incubated with a mouse monoclonal anti-BrdU antibody (Abcam, Cambridge, MA, USA) according to the manufacturer’s instructions to assess the BrdU staining. The hepatocyte proliferation data were obtained by counting the BrdU stained positive hepatocytes (nuclei) in 4 high-power fields (400×) on each slide and are expressed as the mean values. For the PAS staining, after deparaffinization and hydration, the slides were immersed in periodic acid for 8 minutes. The slides were washed with water and subsequently treated with Schiff’s reagent for 15 min, followed by staining with Mayer’s Haematoxylin (Solarbio, Beijing, China).

### Liver function test and circulating cytokine levels

Blood samples were collected via the inferior hepatic vein at designated time points. The plasma samples were isolated, and the levels of aspartate amino-transferase (AST) and alanine aminotransferase (ALT) were measured. The relative cytokine concentrations in the plasma were also detected using the enzyme-linked immunosorbent assay (ELISA) technique following the manual’s instructions (CUSABIO, Wuhan, China). Then, the absorbance of each well was read at 450 nm using a Multiskan microplate reader (Thermo Fisher Scientific, Waltham, MA, USA), and the results were calculated by curve-fitting statistical software (Curve Expert professional 2.6.3, Boston, MA, USA).

### Histological and immunohistochemistry examinations

For the histology and immunohistochemistry analyses, all samples were prepared and stained according to standard protocols^54^. The slices were stained with haematoxylin and eosin (H&E). Briefly, the procedures used for the immunohistochemistry were as follows: after heating at 60 °C for approximately one hour, the slices were deparaffinized in xylene and rehydrated through graded alcohol. Citrate buffers were applied to retrieve the antigens, followed by quenching endogenous peroxidase activity with hydrogen peroxide. The samples were incubated overnight at 4 °C with anti-mouse primary antibodies (CD31, Sino Biological Inc., China; Ki67, Bioss, China) at concentrations of 1:2000 and 1:400. A biotinylated secondary antibody labelled with streptavidin-horseradish peroxidase (ZSGB biotech, Beijing, China) was applied to detect the antibodies through a DAB staining kit (ZSGB biotech, Beijing, China). For the negative control, the primary antigens were replaced by PBS solutions. Two experienced observers carried out the immunohistological staining evaluation in a blinded fashion. The image acquisition and data analysis were performed under a microscope (Olympus, Japan).

### Real-time quantitative reverse-transcriptase polymerase chain reaction (qRT-PCR) assay

The relative mRNA expression levels of different genes were detected using the qRT-PCR technique, and all steps followed the manufacturer’s instructions with proper modifications. Briefly, the total mRNA from 30 mg liver tissues was extracted using TRIzol Reagent (Invitrogen), and 2 μg RNA were quantified for reverse transcription to complementary DNA (cDNA) using a RevertAid First Strand cDNA Synthesis Kit with random decamer primers (Thermo Fisher Scientific). The relative mRNA levels were measured using IL-6, TNF-α, TLR4, MyD88, TSP-1 and TGF-β TaqMan probes and an ABI 7500 thermocyter (Applied Biosystems, USA). Rn18S served as an internal control. The cycling program included an initial denaturalization at 94 °C for 3 minutes, 94 °C for 30 seconds, 58 °C for 30 seconds, and 72 °C for 30 seconds for 35 cycles, and after the final cycle, 72 °C for 7 minutes. For further verification, the products were analysed by agarose gel electrophoresis. The 2^−ΔΔCt^ method was used to determine the specific *Ct* value of each target gene.

### Western blot analysis

The Western blot analysis was carried out using the following steps as previously described^23^. The liver homogenates were prepared using 30 mg liver samples in the appropriate volume (−300 μL) of RIPA buffer (Thermo Fisher Scientific) containing 25 mM Tris, HCl pH 7.6, 150 mM NaCl, 1% NP-40, 1% sodium deoxycholate, 0.1% SDS with the addition of 100:1 (v/v) protease inhibitor cocktail (Thermo Fisher scientific). The protein concentration was assessed using a BCA protein assay kit (Thermo Fisher Scientific). The denaturation of 20 μg protein extracts was followed by separation using SDS-PAGE (Solarbio, Beijing, China) and transferring to PVDF membranes. The samples were incubated with primary antibodies against MyD88 and STAT3 overnight under gentle shaking at 4 °C. The appropriate HRP-linked secondary antibodies were used, and the density of the bands was detected using Image Lab Software 3.0 (Bio-Rad laboratories, USA).

### Statistical analysis

The statistical analysis was performed using the Statistical Package for Social Science (SPSS) version 17.0 (SPSS Inc., Chicago, IL, USA). For continuous data displaying a normal distribution, the values are presented as the *Mean* (*X̅* ) ± *Standard Deviation* (*SD*) and were compared using an *independent t-test*. For skewed data, the values are expressed as the *Median (Interquartile)* and were compared with the *Mann–Whitney U test* or the *Wilcoxon signed-ranks test* as appropriate. Statistical significance was set at the 5% level (*p* ≤ 0.05) and marked as follows: ^*^p ≤ 0.05; ^**^p ≤ 0.01; and ^***^p ≤ 0.001.

## Supplementary information

Supplementary information

## Data Availability

The datasets generated and/or analysed in the current study are available from the corresponding author upon reasonable request.
