# Supplementary material for: Subcutaneous Inoculation of Echinococcus multilocularis Induces Delayed Regeneration after Partial Hepatectomy
Source: Sci Rep. 2019 Jan 24;9:462. doi: 10.1038/s41598-018-37293-0 (PMC6345980; doi:10.1038/s41598-018-37293-0)
Supplement: Supplementary file 1 — Supplementary information [file 41598_2018_37293_MOESM1_ESM.docx]

Supplementary information for:

“**Subcutaneous Inoculation of *Echinococcus multilocularis* Induces Delayed Regeneration after Partial Hepatectomy**”

Shadike Apaer, Tuerhongjiang Tuxun, Heng Zhang, Amina Aierken, Tao Li, Jin-Ming Zhao, Hao Wen.

**Content:**

Figures S1-S2


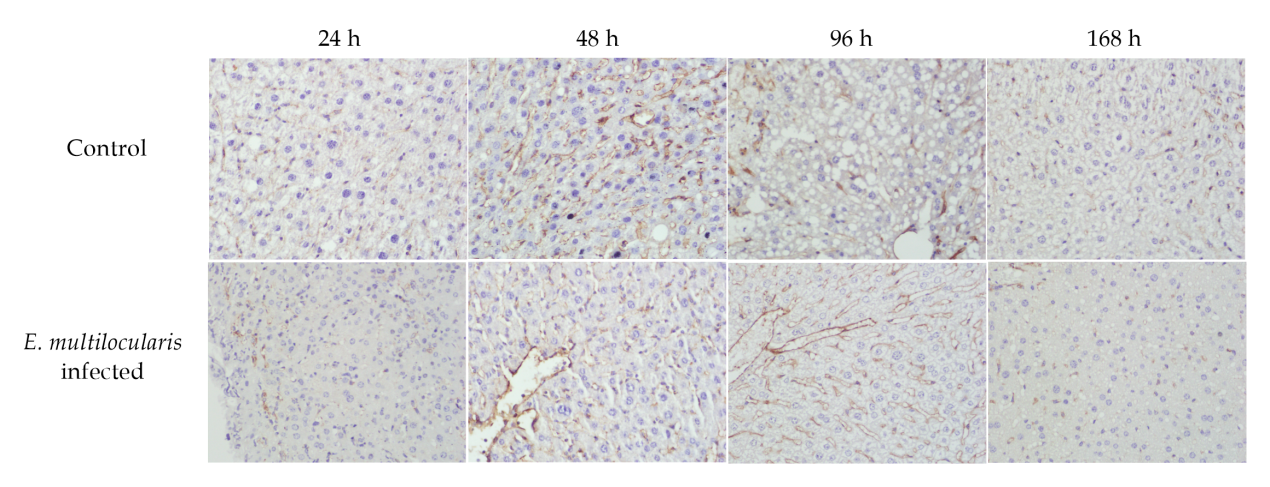


**Figure S1**

Immunohistochemical staining of CD31 in regenerated liver tissues. Representative figures indicated the weak staining at 24 h after surgery both in control and *E. multilocularis* infected mice. Relative higher microvessel density was observed at 48 h and gradually decreased levels after that in control group. Rapid increasing at 48 h and peak density levels at 96 h was found in *E. multilocularis* infected group (Original magnification 400×).


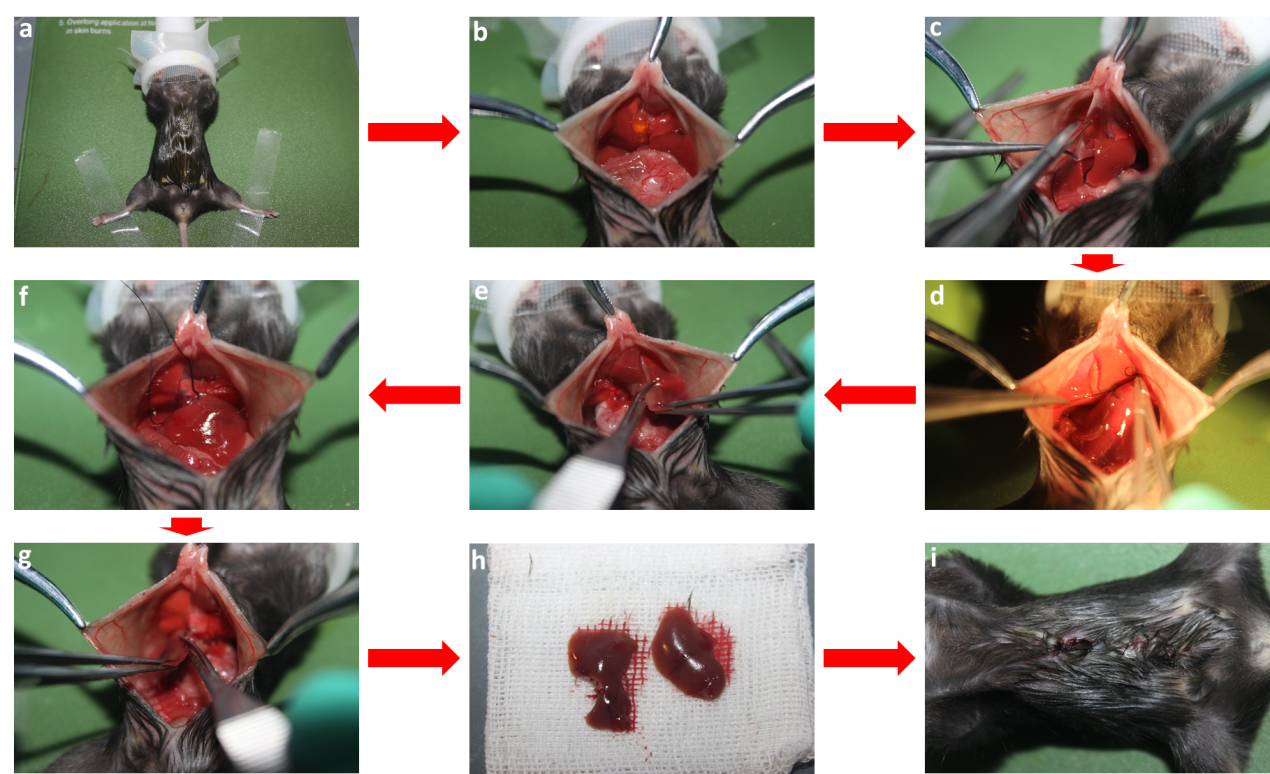


**Figure S2**

Implementation of liver partial hepatectomy (*PHx*). (a) Preparation of experimental mice by fastening on a warming blanket and inducing anesthesia with inhalational isofluorane (2%) and an oxygen flow rate of 2 L/min. (b) Upper midline incision was made. (c) Hepatic ligaments was separated. (d-g) Ligation and resection of left lateral lobe (d-e, 35%), left and right portion of median lobe (f-g, 33%). (h) Resected liver sections. (i) Closure of peritoneum as single layer continuous suture.
